# The Survival of Pear Dormant Buds at Ultra-Low Temperatures

**DOI:** 10.3390/plants10112502

**Published:** 2021-11-18

**Authors:** Alois Bilavcik, Milos Faltus, Jiri Zamecnik

**Affiliations:** Crop Research Institute, Drnovska 507, 16106 Prague, Czech Republic; faltus@vurv.cz (M.F.); zamecnik@vurv.cz (J.Z.)

**Keywords:** cryopreservation, dormant buds, low-temperature resistance, pear tree

## Abstract

Currently, there is a varietal diversity decline in pear orchards of the Czech Republic. Thus, the safe storage of their gene pool collections is becoming increasingly important. Therefore, the ultra-low temperature survival after two-step cryopreservation treatment of dormant buds was tested for a safe and rapid way to conserve pear germplasm in a broader range of varieties. The following varieties crucial for cultivation in the Czech Republic were tested; ‘Amfora’, ‘Beurré Hardy’, ‘Bosc’, ‘Clapp’s Favourite’, ‘Conference’, ‘Dicolor’, ‘Erika’, ‘Lucas’, ‘Williams’ and ‘Williams Red’. In 2011 and 2012, dormant pear buds were dehydrated to 40.1% and 36.0% water content, respectively, before cryopreservation. The average regeneration of the dormant pear buds after cryopreservation by the two-step cryoprotocol in 2011 and 2012 was 54.3% and 16.1%, respectively. The mentioned cryopreservation procedure is suitable for the safe storage of dormant buds in most tested pear varieties.

## 1. Introduction

The number of cultivated varieties of individual fruit trees decreased significantly with the development of intensive orchard management. Only 11 pear varieties are significantly used in the current production of pear trees in the Czech Republic [1]. From a pomological or agrotechnical point of view, pear orchard collections of individual curators are considered old and unattractive. Currently, unsuitable pear varieties are kept in situ in the Czech Republic. These in situ collections are exposed to various adverse effects that negatively affect the number of accessions stored, such as pests, diseases, climate change effects, etc. Therefore, a safe backup of in situ collections is one of the primary tasks of current programs for preserving the genetic diversity of vegetatively propagated crops. Cryopreservation is currently one of the only practical, safe and long-term conservation techniques used as an alternative method to safeguard these species. Pear is an important fruit species not only in the Czech Republic but also in the world. Therefore, there is a need to test its safe storage by cryopreservation and ensure a reliable and secure alternative for the long-term backup of the species.

Cryopreservation is a method of storing plants at ultra-low temperatures. It is a technology enabling long-term storage of biological material at a very low temperature (usually in liquid nitrogen at −196 °C or in liquid nitrogen vapours below −130 °C), while maintaining its viability after transfer to normal conditions [2]. During the cryopreservation procedure, it is necessary to induce processes and conditions in parts of plants that increase their natural resistance to the formation of ice crystals in their tissues and related ice induced dehydration [3,4]. For the cryopreservation of fruit trees, methods based on biological glass formation of in vitro cultures are used [5,6,7]. In addition, the recently reintroduced two-step cryopreservation—mainly in dormant buds of fruit trees are also used [2,8].

The first report of the survival of pear dormant bud in liquid nitrogen was by Sakai and Nishiyama [9]. Oka et al. [10] were able to regenerate plants from cryopreserved dormant buds of *Pyrus serotina* Rehder, ‘Senryo’ by in vitro transfer of meristematic tissues, but achieved a rate of regeneration of less than 8%. Using a similar procedure of in vitro regeneration, Suzuki et al. [11] obtained 88.6% survival of dormant buds of *Pyrus communis* L. ‘Beurre d’Amanlis’ after dehydration to 41% water content. The other 12 cryopreserved pear varieties by Suzuki et al. [11] had survival from 55.5% to 92.5%. However, after they directly micrografted the cryopreserved buds onto rootstocks, the survival was only 30% because of injury of vascular tissues. Guyader et al. [12] adapted the protocol developed at NCGRP, Fort Collins, USA [2,13] with uninodal dormant pear segment pre-freezing at −5 °C until they reached 23% water content. They used slow cooling at a rate of 1 °C h^−1^ to −30 °C and 24 h annealing before placing in vapour phase over liquid nitrogen in a liquid nitrogen freezer. After slow rewarming and rehydration, the chip budding on Kirchensaller pear rootstocks was used. The average regeneration percentage of the 15 cryopreserved cultivars was 26.9%. Additionally, the pre-treatment of dormant buds prior to the two-step cryopreservation with different cryoprotectant solutions was applied. Based on 2,3,5-Triphenyltetrazolium chloride (TTC) staining viability tests, Zhumagulova et al. [14] evaluated six cryoprotective solutions and found that PVS3 solution gave the best survival rates. Recently, forced bud development [15] may also be used as a recovery system if followed by tissue culture regeneration after two-step cryopreservation. This post-cryo recovery approach led to a successful in vitro shoot tip establishment by using an antimicrobial forcing solution (8-hydroxyquinoline citrate and sucrose).

In summary, various methods of cryoconservation of pear according to a type of source material, in vitro or dormant buds, have been published. Cryopreservation of in vitro cultures has its advantages in the possibility of a well-defined treatment on relatively homogeneous plant material, dissected shoot tips. However, these procedures are laborious and time-consuming. When cryopreserving dormant buds by two-step freezing, it is difficult to prepare such homogeneous plant material in terms of size, maturation, or hardening, and this material is only available during a limited winter period. However, a larger number of varieties can be frozen in a relatively short time. Therefore, this work aimed to evaluate a two-step cryopreservation protocol for dormant pear buds with direct regeneration via chip budding onto pear rootstock to contribute to the safe storage of the pear gene pool.

## 2. Results

The average water content of dormant buds of the tested pear varieties in 2011 was 40.1 ± 1.46%. In 2012, pear varieties were freeze-dehydrated to an average water content of 36.0 ± 1.73%. The highest dehydration occurred in 2011 for the variety ‘Lucas’ (37.1%) and the lowest for the variety ‘Erika’ (46.2%). In 2012, the variety ‘Erika’ was the most dehydrated (32.3%) and the variety ‘Conference’ the least dehydrated (37.9%). Thus, the dehydration of pear varieties in 2012 was 4.1 ± 2.59% higher than in 2011. The highest difference in dehydration was in the variety ‘Erika’ (9.2%) and the lowest in ‘Amfora’ (1.0%), see Table 1.

The average regeneration of pear dormant buds after cryopreservation by a two-step cryoprotocol in 2011 was 54.3 ± 23.71%. The highest regeneration showed ‘Clapp’s Favourite’ (83.3%) and the lowest ‘Williams Red’ (8.3%). In 2012, the average regeneration of pear varieties was 16.1 ± 12.33%. The highest regeneration showed ‘Clapp’s Favourite’ (37.5%) and the lowest ‘Erika’ (0%). There was a high degree of variability in regeneration percentage between the two years: the varieties ‘Lucas’ and ‘Conference’ showed the most significant difference between the two years. Although ‘Williams Red’ and ‘Dicolor’ showed the least difference between the two years, they also showed the worst regeneration in 2011. ‘Bosc’ and ‘Erika’ showed a smaller decrease in regeneration percentage, but they both fell to zero in 2012. On average, regeneration of 37.8 ± 17.87% was achieved in dormant pear buds after cryopreservation in both years, see Table 2.

**Figure 1:**
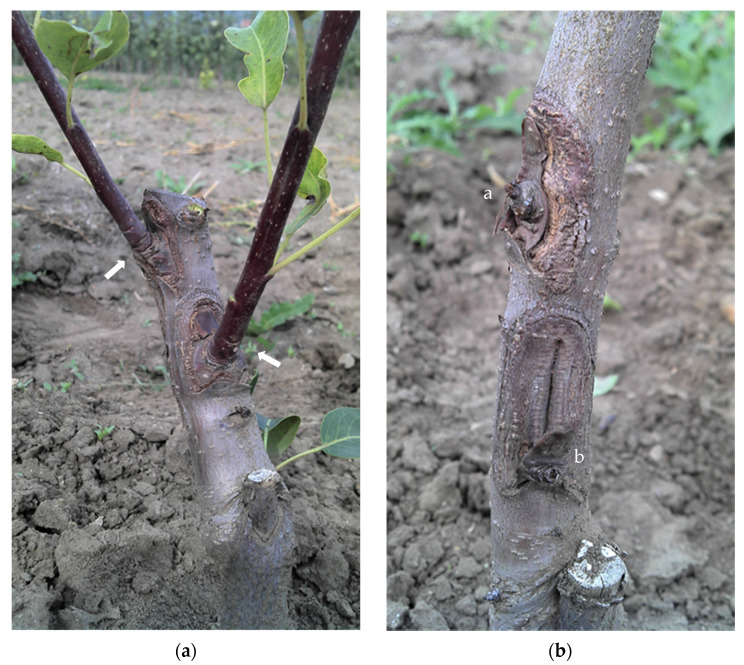
Regeneration of dormant buds of pear variety ‘Clapp’s Favourite’ after two-step dehydration cryopreservation. The grafting was done by chip budding in the spring period: (**a**) Arrows show the dormant buds sprouted in new shoots from meristematic parts of the chip; (**b**) the upper bud (**a**) survived but did not sprout into a new shoot. The bud sprouted into a new shoot at the second sap during the summer period. The lower bud (**b**) did not survive and was released. The buds were chip budded on pear seedlings in an orchard during the spring sap.

Survival and regeneration of dormant pear buds after two-step dehydration cryopreservation is presented in Figure 1.

## 3. Discussion

A two-step cryopreservation protocol that includes an initial dehydration step is important to reduce ice crystal formation in tissues and increase survivability and regeneration. Within both years of the study, the difference in the dehydration degree of the varieties used was kept to a minimum (SD up to 1.7%). In the first experiments carried out in 2011, the dehydration level of dormant buds was set to the 40% level [11]. The aim of higher dehydration in 2012 compared to 2011 was to approach the values used for apple trees [16] and comparable levels of pears [12], to reduce the lethal effect of ice crystal formation in tissues and thus to increase regeneration [17]. Both of the above-mentioned authors [12,16] used the dehydration level of 30%. According to our preliminary studies, the level of 30% dehydration was too low, so in 2012 we decided to choose dehydration at approximately half the level, close to 36%. We maximally standardized the sampling and preparation of the dormant buds and their cold hardening conditions so that they were as similar as possible in both years. The regeneration of dormant buds differed significantly from year to year. In 2011, the regeneration of all varieties except ‘Williams Red’ was over the acceptable rate (29%). In the following year (2012), when the dormant buds were intentionally more dehydrated, the regeneration decreased significantly in all varieties. In 2012, regeneration decreased the most for the varieties that showed the highest regeneration in 2011, ‘Lucas’, ‘Conference’, and the least for the low-regeneration varieties, ‘Williams Red’ and ‘Dicolor’. For ‘Erika’, which was the most dehydrated of all varieties (32.3%), regeneration fell to zero in 2012.

A similarly significant decrease in the regeneration of dehydrated dormant pear buds below 34% was found by Suzuki et al. [11]. Regeneration of dormant pear buds frozen in 2011 reached (except for two varieties (‘Williams Red’ and ‘Dicolor’)) similar values to Suzuki et al. [11]. These authors achieved regeneration in 12 pear varieties in the range of 55–92%. The dormant bud regeneration procedure may have caused their slightly higher values of regeneration. They dissected shoot tips from cryopreserved dormant buds and regenerated them in vitro. In vitro conditions can be better standardized for regeneration, and it is possible to eliminate adverse conditions compared to regeneration by chip budding in an orchard. Differences in the regeneration of the same varieties in different years were also found in fruit trees by other authors, such as Höfer [18]. These differences can be justified by the differences in seasons and the response of varieties to them [2], if there is not precise pre-treatment of plant material by cold hardening at below zero temperatures [13]. Jenderek et al. [19] tested if the physical geographic location of the apple dormant buds, and by interference the preharvest temperature, compromised cryotolerance. Their data showed that for three locations tested, the geographic location of the apple dormant bud harvest did not adversely affect the bud cryopreservability. They also did not find a significant difference in cryopreservability of tested apple varieties in two from three seasons and the only different season was probably caused by an equipment malfunction. When using the same protocol in different series of tests at different dates in one season, Guyader et al. [12] found regeneration after cryopreservation fluctuating from 11.1% to 91.7% for pear variety ‘Williams‘ and they identified several factors, which seemed to significantly influence the results: bud morphotypes, rehydration phase (technique used and duration), rootstock calibre, grafting technique, etc. On the other hand, an internal physiological state, such as endodormancy, does not affect the cryopreservability of dormant buds themselves [20]. The above publications led us to believe that by maintaining the same conditions for sampling, pre-treatment, hardening, and post-cryopreservation regeneration we could separate the extent of dehydration in different years. A comparison of published cryopreservation results of selected pear varieties (*Pyrus communis* L.) with the results obtained in this work is in Table 3. The first report on cryopreservation of pear varieties ‘Amfora’, ‘Dicolor’, ‘Erika’, and ‘Williams Red’ is presented. With the exception of the varieties ‘Bosc’ and ‘Williams’, we achieved higher regeneration in all comparable varieties. On average, the varieties frozen by encapsulation-dehydration of in vitro cultures achieved a 40% reduction in regeneration compared to our results [21,22,23]. The only higher regeneration of cryopreserved in vitro variety, ‘Bosc’, was obtained by controlled two-step freezing of shoot tips pre-treated with a cryoprotectant mixture (polyethylene glycol, glucose, and DMSO) [24]. The DMSO as a cryoprotectant was used by Dereuddre et al. [25] for ‘Beurré Hardy’ with 60% regeneration compared to our 79%. Due to the potential mutagenic effects of the cryoprotectant DMSO [26], there have been attempts to omit the DMSO during the cryopreservation process. A slightly lower average regeneration, by 10% compared to our results was achieved by Guyaeder et al. [12] with a similar method, see Table 3. On the other hand, the two-step freezing cryopreservation of dormant buds modified by introducing sprouting shoots from dormant buds into in vitro had 20% less regrowth in one comparable variety [27], see Table 3. Although the above method was less reliable, it could have potential in a regeneration system eliminating the environmental risk of grafting cryopreserved buds under orchard conditions.

According to our results, the optimal dehydration of dormant bud cryopreservation of selected pear varieties by the two-step freezing was 40%. It can be concluded that the tested procedure of cryopreservation of dormant pear buds can already be used and, after optimizing the dehydration conditions to extend it to hitherto less cryopreservable varieties.

## 4. Materials and Methods

One-year-old dormant shoots of the pear varieties ‘Amfora’, ‘Beurré Hardy’, ‘Bosc’, ‘Clapp’s Favourite’, ‘Conference’, ‘Dicolor’, ‘Erika’, ‘Lucas’, ‘Williams’ and ‘Williams Red’ were used in the experiments. Shoots were taken from outdoor conditions during January 2010 and 2011 from the orchards of SEMPRA Litomerice Ltd., Litomerice, Czech Republic (USDA Plant Hardiness Zone 7a). The shoots were cut into uninodal segments with one bud in the middle and placed in a freezer at −4 °C. At this temperature, they were freeze-dehydrated for 4–7 weeks. The water content was determined gravimetrically on a fresh weight basis after drying a random sample of 5 segments at 85 °C for constant weight. In the first year, the dehydration level was set at 40% and values below 38% of the water content in the second year. After drying, a sufficient number of nodal segments (from 20 to 25 segments) were frozen in 50 mL tubes covered with aluminium foil, Kartell Conical Grad Test Tube, Kartell S.p.A., Italy, with a two-step cryoprotocol. In the first step of the cryoprotocol, the temperature was lowered from −4 °C to −25 °C (cooling rate 2 °C h^−1^) in a computer-controlled freezer, Arctiko LTF 325, Denmark, and after equilibration for 12 h; the second step of the cryoprotocol was done; the tubes were immersed in liquid nitrogen. At least 120 buds from each variety were frozen for the cryobank storage, LS4800 Taylor Wharton, USA, and 24 buds for evaluation of control sample regeneration. Buds for survival evaluation of the cryoprotocol were placed at +4 °C and allowed to slowly thaw spontaneously. After 2 weeks of rehydration of the buds in moist white peat, Baltic white peat, Hawita, Germany, at +4 °C, the buds were chip budded on pear seedlings, *Pyrus communis* L., in an orchard during the spring sap. After approximately two months, the survival of the buds was assessed. The sprouted buds were evaluated as regenerated and successfully cryopreserved, and the non-sprouted buds were evaluated as unregenerated and damaged by cryopreservation.

## 5. Conclusions

The paper presents the cryopreservation procedure of dormant pear buds tested in a broader range of varieties. It is evident that the successful cryopreservation of dormant pear bud depends both on the variety and, especially, on the acclimation of the buds. The acclimation not only depends on the specific course of the winter season, and thus, on the naturally induced frost resistance of dormant buds, but also on the cryopreservation procedure with artificial frost dehydration. According to the results, the frost dehydration to 40% of water content enabled successful cryopreservation for most pear varieties. The obtained results show the potential of introducing the tested cryopreservation procedure in the cryobanking of pear species, thus ensuring the safe backup of endangered in situ pear collections.

## Figures and Tables

**Table 1:** The water content of dormant pear buds after freeze-drying before a two-step cryopreservation cryoprotocol. The water content of varieties in 2011, 2012, the average water content and the difference between 2011 and 2012 is shown.

Variety			Water Content (%)	
	2011	2012	Average	Δ 2011–2012
‘Amfora’	38.7	37.7	38.2	1.0
‘Beurré Hardy’	40.5	34.5	37.5	6.0
‘Bosc’	38.3	37.2	37.7	1.1
‘Clapp’s Favourite’	40.8	37.9	39.4	2.9
‘Conference’	40.8	37.9	39.3	3.0
‘Dicolor’	41.5	35.0	38.2	6.6
‘Erika’	41.5	32.3	36.9	9.2
‘Lucas’	37.1	35.6	36.3	1.5
‘Williams’	40.8	37.1	38.9	3.8
‘Williams Red’	41.2	35.3	38.3	5.8
Average	40.1	36.0	38.1	4.1
SD	1.46	1.73	0.95	2.59

**Table 2:** Regeneration of dormant pear buds after a two-step dehydration cryoprotocol.

Variety				Regeneration (%)	
	2011	SD	2012	SD	Δ 2011–2012
‘Amfora’	65.8 ^cde^	15.9	29.2 ^cd^	11.79	36.2
‘Beurré Hardy’	79.2 ^de^	5.9	29.2 ^cd^	5.89	48.7
‘Bosc’	33.3 ^ab^	21.2	0.0 ^a^	0.00	33.3
‘Clapp’s Favourite’	83.3 ^de^	11.8	37.5 ^d^	10.21	45.8
‘Conference’	71.7 ^cde^	24.6	12.5 ^abc^	10.21	56.7
‘Dicolor’	29.2 ^ab^	15.6	16.2 ^abc^	15.73	13.2
‘Erika’	46.7 ^bc^	11.2	0.0 ^a^	0.00	46.2
‘Lucas’	75.8 ^cde^	18.3	11.1 ^abc^	15.71	64.9
‘Williams’	50.0 ^bcd^	10.2	20.8 ^bcd^	5.89	29.2
‘Williams Red’	8.3 ^a^	5.9	4.2 ^ab^	5.89	4.0
Average	54.3		16.1		37.8
SD	23.71		12.33		17.87

^a–e^ averages with the same index do not differ significantly (α = 0.05, analysis of variance—LSD test).

**Table 3:** Reports on cryopreservation of selected pear varieties (*Pyrus communis* L.) and comparison with the obtained results presented in this work (dormant bud, two step-freezing, slow-cooling prior to storage in LN).

Variety	Max. Regrowth ^a^ (%)		Published Results		
		Max. Regrowth [%]	Source ^b^	Method ^c^	Ref.
‘Amfora’	66				
‘Beurré Hardy’	79	40	iv	En-Dehy	[21]
‘Beurré Hardy’	79	60	iv	DMSO/TSF	[25]
‘Bosc’	33	90	iv	PGD/TSF	[24]
‘Clapp’s Favourite’	83	14	iv	En-Dehy	[22]
‘Clapp’s Favourite’	83	~33	db	Dehy-TSF-Graft	[12]
‘Conference’	72	50	db	Dehy-TSF-Graft	[12]
‘Dicolor’	29				
‘Erika’	47				
‘Lucas’	76	44	iv	En-Dehy	[22]
‘Williams’	50	26	iv	En-Dehy	[23]
‘Williams’ ^d^	50	~30	db	Dehy-TSF-iv	[27]
‘Williams’	50	92	db	Dehy-TSF-Graft	[12]
‘Williams Red’	8				

^a^ The obtained results presented by this work. Freeze dehydration followed by two-step freezing, slow cooling prior to storage in LN, regeneration by grafting (Dehy-TSF-Graft). ^b^ Type of the cryopreserved plant tissue, iv = shoot tips from in vitro culture, db = nodal segments with dormant buds. ^c^ En-Dehy = encapsulation–dehydration; DMSO/TSF = DMSO pre-treatment followed by two-step freezing, slow cooling prior to storage in LN; PGD/TSF = PGD cryoprotectant mixture pre-treatment followed by two-step freezing, slow cooling prior to storage in LN, Dehy-TSF-Graft = freeze dehydration followed by two-step freezing, slow cooling prior to storage in LN, regeneration by grafting; Dehy-TSF-iv = freeze dehydration followed by two-step freezing, slow cooling prior to storage in LN, regeneration by in vitro. ^d^ The ‘Bartlett’ pear in the United States and Canada.

## Data Availability

The data presented in this study are available on request from the corresponding author.
